# Gender-Specific Impact of Sex Hormones on the Immune System

**DOI:** 10.3390/ijms24076302

**Published:** 2023-03-27

**Authors:** Francesca Sciarra, Federica Campolo, Edoardo Franceschini, Francesco Carlomagno, Mary Anna Venneri

**Affiliations:** Department of Experimental Medicine, Sapienza University of Rome, 00161 Rome, Italy

**Keywords:** androgens, estrogens, progesterone, sex hormones, granulocytes, lymphocytes, immune system

## Abstract

Sex hormones are key determinants of gender-related differences and regulate growth and development during puberty. They also exert a broad range modulation of immune cell functions, and a dichotomy exists in the immune response between the sexes. Both clinical and animal models have demonstrated that androgens, estrogens, and progestogens mediate many of the gender-specific differences in immune responses, from the susceptibility to infectious diseases to the prevalence of autoimmune disorders. Androgens and progestogens mainly promote immunosuppressive or immunomodulatory effects, whereas estrogens enhance humoral immunity both in men and in women. This study summarizes the available evidence regarding the physiological effects of sex hormones on human immune cell function and the underlying biological mechanisms, focusing on gender differences triggered by different amounts of androgens between males and females.

## 1. Introduction

The immune system protects the body against infections similarly in men and women, although women display stronger innate and adaptive immune responses than men [1], making them less susceptible to microbial infections and able to fight viruses in a more efficient way [2]. On the other hand, women are also more frequently affected by autoimmune disorders, such as systemic lupus erythematosus, multiple sclerosis, and rheumatoid arthritis [3]. The reasons behind these sex differences in the immune system are a topic of current study and gender difference therefore needs to receive more attention. Experimental and clinical data indicate contributing factors such as genetics and hormonal influences [1,4]. Indeed, the hormonal interactions among gonadal steroids, adrenal glucocorticoids, growth hormone (GH), and prolactin lead to immunological dimorphism in the sexes [5]. Sex hormones include androgens, estrogens, and progestogens that, besides controlling reproduction, influence both physical and mental health and well-being [6,7]. Their importance was recently evidenced in the context of the COVID-19 pandemic with the identification of the role of testosterone (Te) in modulating cytokine release and determining disease severity and outcome [8,9].

**Figure 1:**
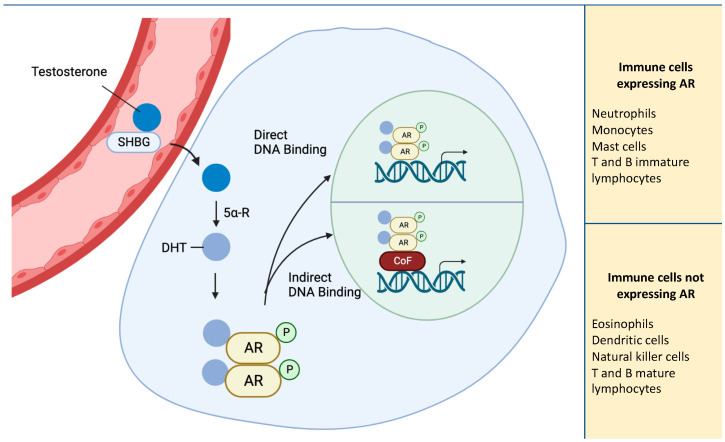
Schematic representation of AR pathway. AR activation by Testosterone, converted to DHT via 5α-reductase causes receptor dimerization and translocation to the nucleus. In the nucleus, AR binds to DNA directly or through cofactors. Eosinophils, DCs, NKs, and both T and B mature lymphocytes do not express AR and the actions of androgens on these cell types are indirectly mediated. Abbreviations: 5α-R, 5α-reductase; AR, androgen receptor; CoF, cofactor; DHT; dihydrotestosterone; P, phosphorylation; SHBG, sex hormone-binding globulin; Testosterone.

Sex hormones play a role in modulating immune cells, influencing: (1) the differentiation from common progenitors cells, (2) lifespan and survival, as well as (3) their capacity to respond to infections and malignancies (e.g., with regards to phagocytic activity, cytokine secretion, production of antibodies) [10]. Androgens represent a group of gonadal and adrenal-derived hormones, and the most biologically relevant androgens are Te, dihydrotestosterone (DHT), Δ^4^-androstenedione (Δ^4^), and dehydroepiandrosterone sulfate (DHEA-S). The biological actions of androgens are mediated by the androgen receptor (AR), a member of the steroid hormone nuclear receptor family, and a ligand-dependent nuclear transcription factor [11]. Androgens act on target tissues via the AR in a DNA binding-dependent manner, regulating the transcription of target genes, or in a DNA binding-independent manner, triggering phosphorylation of second messenger signaling cascades (Figure 1) [12]. The evidence for the effects on AR-negative immune cell types has led the hypothesis that androgens can also exert indirect actions, possibly mediated by other mechanisms through which steroid hormones exert their effects on immune cells. For example, progesterone can exert its action on immune cells by binding to the other receptor. An alternative mechanism that can be used by sex hormones can depend on their lipophilic nature, which can integrate into the membrane exerting a function on immune cells. Further studies are needed to elucidate the mechanisms of interaction between immune cells and steroid hormones [13].

ARs are expressed in several tissues, mainly in the reproductive organs [14] and also in both myeloid and lymphoid-derived immune cells [15]. They have been identified in the bone marrow (BM) of men and women, and in stromal cells, endothelial cells, macrophages, and myeloid precursors [16].

Estrogen receptors (ERs) regulate both the innate and adaptive immune pathways in response to circulating estrogen levels. Each of the three ERs (ERα, ERβ, and the G protein-coupled receptor) play a role in immune cell development, differentiation, and function [17], besides regulating the main physiological processes in the reproductive, endocrine, nervous, skeletal, and cardiovascular systems in both women and men [18].

The expression of progesterone receptors (PRs) in immune cells, although not ubiquitous [19], indicates an involvement of progesterone (P4) in both the innate and adaptive immune systems. Two significant isoforms of PR, PRA and PRB, mediate most of the physiological functions in humans. Both are present in the endometrial epithelium during the proliferative phase of the menstrual cycle, and their expression increases linearly with estrogen levels. The PRs are also expressed in the pituitary, hypothalamus, testis, epididymis, prostate, and mammary glands [20].

Androgens and P4 mostly promote immunosuppressive or immunomodulatory effects, whereas estrogens enhance the humoral immunity both in men and in women [1].

The aim of this review is to summarize the available findings on the effects of sex hormones on human immune cells, both on the innate and adaptive components of the immune system focusing on the sex differences in immune responses.

## 2. Innate Immune Cellular Components

### 2.1. Neutrophils

Neutrophils, alongside their hematopoietic progenitor cells, express ARs and androgens exert a stimulatory role with regards to their differentiation [16], granulopoiesis, and cytokine production [13,21]. Both ERs and PRs are also expressed in neutrophils and contribute to regulate neutrophilic functions [17,22]. In women, the spontaneous apoptosis of the neutrophils is markedly delayed compared to men, and the administration of estradiol (E_2_) and progesterone (P_4_) further increases the lifespan of neutrophils in males and females (Table 1) [23]. A differential maturation status is also present, with a more immature phenotype observed in men compared to women, which translates into an altered response to cytokine stimulation, and a decreased ability to form neutrophil extracellular traps (NETs) [24].

Female sex hormones (estrogens and progestogens) act at the mitochondrial level, reducing cytochrome C (Cyt C) export, and thus cleavage and caspase activity, leading to increased peripheral concentrations [25]. In addition, female sex hormones enhance the primary bactericidal mechanism in neutrophils via the intracellular production of reactive oxygen intermediates (ROI), such as superoxide radicals. Differences in neutrophils exist not only between the sexes, but also among women in different physiological conditions, being higher in fertile women both during the luteal phase and during pregnancy [13] (Table 1). Neutrophils are influenced by the human chorionic gonadotropin (hCG) produced by the placenta trophoblast, which binds the LH/CG receptor (LH/CGR), conferring fetal tolerance by: 1) promoting neutrophilic invasion of the decidua, placenta, and fetal membranes, 2) inducing a specific regulatory T (T-reg) subpopulation with proangiogenic properties, and 3) promoting NETs formation to protect the embryo [26]. Instead, low concentrations of hCG inhibit proliferation and induce apoptosis in human neutrophils by inhibiting neutrophilic function and activation [27].

**Table 1:** Biological and clinical effects of androgens on immune cell types.

	Biological/Clinical Effects		Hemogram	Ref.
Cell Type	Female	Male		
**Neutrophils**	↑ Apoptosis	↓ Maturation	↑ Concentration	[13,25,28]
**Monocytes**	↓ Pro-inflammatory cytokines	↑ Differentiation	↑ Concentration	[29,30]
**Macrophages**	↓ Pro-inflammatory cytokines	↓ Pro-inflammatory cytokines	NA	[15,31]
	↑ M2 polarization	↓ M1 polarization	NA	[32]
**Natural killer**	/	/	No effects	[29]
**Dendritic cells**	↑ IFNα production	↓ Pro-inflammatory cytokines ↑ CD16^+^ in hypogonadic	No effects	[29,33,34]
**B lymphocytes**	↓ CD5^+^ immature/transitional B cells ↑ memory B cells	Correlation between DHT levels at birth and CD5^+^ B cells number ↑ B cells lymphopoiesis	No effects	[29,35,36,37]
**T lymphocytes CD4**	↓ CD4^+^ T cells in postmenopausal	↓ CD4^+^ T cells subpopulation in gonadal castration	No effects	[29,38]
**Th1/Th2**	↑ Th1	↑ Th2	/	[38]
**T-regs**	↑ in treated adrenal insufficiency patient	↑ T-regs		[39,40,41]
**T lymphocytes** **CD8**	↑ CD8^+^ T cells in postmenopausal females	/	No effects	[29,42]

Abbreviations: NA, not applicable.

Androgen deprivation therapy (ADT) in patients affected by castration-sensitive prostate cancer leads to neutropenia [43] and to an aberrant neutrophilic phenotype, with more banded and immature neutrophils [28]. Furthermore, hypogonadal men treated with Te show an increased number of neutrophils (Table 1) [30] with a reduction in superoxide anion and lipid peroxidation, and an increase in nitric oxide concentrations [44].

Androgen-mediated immunosuppressive effects have been described with regards to cytokine production in males: hypogonadal men treated with Te replacement therapy show suppression of the neutrophilic proinflammatory cytokines Tumor Necrosis Factor alpha (TNF-α), Interleukin 6 (IL-6), and Interleukin 1 beta (IL-1β), and augmented anti-inflammatory cytokine Interleukin 10 (IL-10) [45]. The androgens show a proliferating effect on the neutrophils not only in men, but also in the context of polycystic ovary syndrome (PCOS)-affected women, a condition characterized by hyperandrogenism, which frequently features neutrophilia [46]; interestingly, the use of anti-androgens (e.g., flutamide) reverses the effects on the neutrophilic count [47].

### 2.2. Monocytes

Monocytes express ARs, ERα (and to a minor extent ERβ), as well as PRs [48,49], and thus sex hormones may exert direct modulating effects on this cell type [15,16].

Immune sex differences have been observed in the development of the myeloid lineage: men show greater BM monocyte differentiation with an increased blood concentration compared to women. These differences are partly attributable to AR-androgen signaling, which, even if not influencing the total BM cell numbers, directly affects monocyte development, and modulates the turnover of mature blood monocytes [50,51]. In vitro studies show an increase in IL-1β and IL-12 in male compared with female monocytes treated with Te [51], and a reduction in IL-6 production [45]. Collectively, these data suggest that androgens have an effect in determining the production of cytokines in the monocytes differently in male and female.

Te replacement therapy in hypogonadal men is shown to increase the concentrations of circulating monocytes [52,53]. These results also suggest an androgen-mediated stimulation of the differentiation in the myeloid lineage progenitor cells [30]. Moreover, sex differences are also detected in the release of monocyte cytokines: the production of a major pro-inflammatory cytokine Interleukin 12 (IL-12) and IL-1β monocytes is detected in men with respect to women [51] (Table 1). In addition, the secretive activity in both male and female monocytes is influenced by androgens: the chronic administration of Te in hypogonadal type II diabetes mellitus (T2DM) patients causes the reduction, or complete abrogation, in the secretion of the monocyte pro-inflammatory cytokines IL-6, IL-1β, and TNF-α [29].

On the other hand, by studying the effects of the administration of estrogens in vivo, it is possible to determine how women using contraceptives containing estrogens and progestogens show a decrease in the production of the pro-inflammatory cytokines interferon gamma (IFN-γ) and TNF-α by monocytes. In addition, an in vivo study suggested how sex hormones are only partly involved in the IL-6 monocytic cytokine production difference between men and women [54].

### 2.3. Macrophages

Macrophages express ARs [55], as well as all ERs, and the PR [48,56]. As such, sex hormones are able to exert direct regulating effects on this cell type.

Human macrophages are subject to hormonal modulation effects. In vitro Te administration exerts an anti-inflammatory action, determining a reduction in the expression of pro-inflammatory cytokines TNF-α and IL-1β (whereas IL-6 is not affected). Androgens reduce the polarization towards the M1 macrophages regulating the inflammatory pathways [15].

On the other hand, estrogens act via ERα to determine an increase in M2 gene expression and polarization [32], although E_2_ administration does not affect their concentrations [31] (Table 1).

Furthermore, DHT is able to induce the cytotoxic capacity of the macrophages to directly target and kill cells in a concentration-dependent manner [57], and DHT is able to switch the macrophage’s phenotype towards M1 polarization [57].

### 2.4. Natural Killer Cells

NK cells are reported to express high levels of ERα in most of the subpopulations examined and, also PRs in some [58]. Of note, NK cells do not express ARs [16] and androgens interact without direct binding to DNA.

The in vivo administration of DHEA to postmenopausal women with adrenal androgen deficiency is shown to increase the circulating CD8^+^/CD56^+^ NKs and decrease the CD4^+^ lymphocytes, also inhibiting the T cell mitogenic and IL-6 responses, the two principal exogenous signals that induce the proliferation and differentiation of T cells [59]. However, the most distinctive NK gender difference is evidenced by their role in pregnancy. This is due to large granular lymphocyte cells (LGL), which are abundant in the endometrium, expressing an NK-like phenotype. LGL increase in number during the first trimester reaching up to almost 70–80% of all endometrial leukocytes, and decreasing afterwards [60] (Table 1). The most abundant lymphocytic subpopulation in the uterus is represented by CD56^+^ NKs, which accumulates in the decidual tissues and differentiates into decidual NKs (dNKs). In vitro and in vivo experiments show how dNKs modulate trophoblast invasion by producing IL-8, CXCL10, and various vascular angiogenic factors [61]. Uterine NK levels also change under different physiologic states, e.g., during the menstrual cycle, with an increase in the proliferative phase, reaching a maximum in the late secretory phase. These differences are influenced by an increase in E2 concentrations, and also by gonadotropins, such as human chorionic gonadotropin (hCG) and luteinizing hormone (LH) [62]. Uterine NK cells do not express progesterone receptors but their function is indeed affected by progesterone [63].

### 2.5. Eosinophils

The AR is not expressed by eosinophils [16], although androgen modulation influences their characteristics by controlling their infiltration into tissues [64]. ERα and Erβ are expressed in eosinophils and are involved in the pro-inflammatory process [65]; also PR was identified in eosinophils [66].

Female patients affected by severe hirsutism and treated with anti-androgenic drugs develop dyspnea with a restrictive ventilatory defect. The bronchoalveolar lavage shows an increased number of eosinophils, of CD8^+^ T cells, and of neutrophils, which disappear after anti-androgenic drug withdrawal [67]. PCOS-affected patients, with pathologically increased androgenic concentrations, are affected by low-grade chronic inflammation in the peripheral blood and ovaries, with elevated peripheral eosinophilic granulocytes, lymphocytes, and monocytes [68].

These data point toward a correlation between the concentration of androgens and eosinophilic influx, and also to an involvement of androgens in anti-inflammatory effects [64] (Table 1).

### 2.6. Mast Cells

MCs express ARs, Erα, and PRs [69,70]. The hormonal androgenic influence on MCs is complex: some studies show how their effects depend not only on sex, but also on the tissues from which the cells were isolated. MCs isolated from different tissue samples, such as men’s foreskin and women’s breast skin, express ARs, although women’s cells express lower AR levels than men [71] (Table 1). In vitro human MC E_2_ treatment has determined both an enhanced degranulation [70] and a reduced secretion of the pro-inflammatory cytokines IL-6 and TNF-α. Conversely, treatment with Te did not influence these processes.

Perinatal androgens can have a significant impact on MC development [72] and an in vitro experiment showed the subsistence of hormonal dose-dependent effects (direct, synergistic, or inhibitory) [71]. A possible mechanism to explain the sexually dimorphic response of MCs to steroids is that sex steroids can activate the intracellular signaling pathways in MCs in a sex-dependent manner [72]. Thus, it will help to understand many MC-related pathophysiological alterations such as asthma and other allergic and inflammatory diseases [73], which have a different prevalence in women compared to men.

### 2.7. Dendritic Cells

Dendritic cells (DCs) derive from common dendritic progenitor cells, which can develop both into myeloid-derived DCs (mDCs) or lymphoid-derived (pDCs) cells. mDCs do not express ARs [74], and to date, there is no evidence about the expression of pDCs and ARs in common dendritic progenitor cells. Androgens interact with DCs without directly binding to DNA, similar to NKs. Decidual DCs express PRs and are highly responsive to high local concentrations of progesterone [63]. Estrogen enhances the differentiation of immature DCs into mature functional DCs [75].

Studies have determined how androgen hormones exert an influence on male human DC subsets, although such pathways are still not well clarified [76]. The effects of androgen (Te) withdrawal on the production of the pro-inflammatory cytokines in pDCs (IL-6, IL-1β, TNF-α) was analyzed in a male population affected by T2D with partial androgen deficiency: the reduction, or complete abrogation, in both spontaneous and ex vivo pro-inflammatory cytokine production was detected, with respect to the controls [29] (Table 1). The distribution and functional status of various peripheral blood DC subsets was also compared, and no statistically significant differences were detected between various DC groups, even if the hypogonadal patients showed a slightly higher number of CD16^+^ DCs that express the activation/degranulation-associated marker CD107b. It was detected how CD16^+^ number reaches statistical significance after inflammatory stimulation (in vitro CpG nucleotide DNA sequence), also highlighting a CD107b inverse correlation with Te, compared to the controls (Table 1) [34]. A similar inverse correlation was determined for LH and follicle-stimulating hormone (FSH), highlighting how some DC subgroups are influenced by their action [34]. To deepen the correlation between Te and CD107b, the effects of androgen replacement were checked on the DCs of hypogonadal men (both mDCs and pDCs) and it was found that Te replacement determined the overexpression of CD107b by the DCs [77].

The DCs also show immune gender differences, both in early infancy and adulthood. The male pDCs IFN-α production responses to the TLR7/8 agonist R-848 challenge are lower than the female responses in early infancy, and such a difference may be attributed to the male surge of androgens during the first 6 months of life (Table 1) [33]. To explain microbial and viral infections, different pathogenic responses in male and female children are also proposed with an involvement of the pDCs-mediated TLRs response. The androgenic influence on human pDCs is confirmed by the in vitro treatment of DHT, which shows a relevant reduction in IFN-α expression [33]. The pDCs in adults show immune gender differences, including peripheral blood leukocyte stimulation by TLR7, which induce higher IFN-α production in women than in men [78]. They display a correlation with the pathogenesis of auto-immune diseases, whose incidence is higher in women compared to men. The studies on systemic lupus erythematosus (SLE) highlight a relevant role for pDCs and IFN-α production [79]. Another relevant connection is found between sex differences in pDCs and pathogenesis in the HIV-1 single-stranded viral infection. The activation of pDCs via the elevated expression of TLR7 and IFN-α, is prognostic indicator for the clinical progression of HIV-1. The pDCs in women are detected to produce more IFN-α responding to the HIV-1 infection and stronger CD8^+^ activation than the male pDCs [80]. 

## 3. Adaptive Immune System

### 3.1. B Lymphocytes

Mature B lymphocytes do not express ARs on their surface [76], and Te treatment does not influence their peripheral blood concentration in humans [30]. However, immature B cells (both pro-B and pre-B stages) express ARs [76], (Table 1), suggesting how an androgen-mediated action can influence the development of immature B cells, rather than acting on mature lymphocytes [76]. Estrogen controls B cell differentiation, activity, function and, unlike CD4 cells, B lymphocytes have more ERβ than Erα [75]. B lymphocyte development occurs in the intersinusoidal spaces of the BM in association with a sessile population of stromal cells, multipotent stem cells located within BM stroma, which give rise to osteoblasts and adipocytes, and secrete a variety of cytokines that affect lymphocyte growth and differentiation. Importantly, BM stromal cells express ARs [16].

Gender differences are observed in human B cell subsets, such as CD5^+^ B cells (one-fifth of normal peripheral blood B cells in early adulthood): a prospective study in 3–8 year-old children showed that female have lower concentrations in CD5^+^ and a higher number of memory phenotype B cells than male [81]. In addition, CD24^hi^CD38^hi^ B cells, immature transitional B cells that, in normal individuals exert suppressive effects by IL-10 production but are quantitatively altered and/or functionally impaired in individuals with various autoimmune diseases, resulted in lower levels in young women compared to men [35]. Furthermore, a positive correlation between DHT levels at birth and higher CD5^+^ B cells is determined in men in comparison to women [82](Table 1).

Adult men and women show relevant gender differences in their B cells. Men affected by rheumatoid arthritis, showing low Te levels as pathological markers, present an enhanced B cell lymphopoiesis [83]. In addition, prostate cancer-affected patients subjected to ADT show an enhanced B cell lymphopoiesis [84]. Furthermore, men, with higher serum Te levels, have lower antibody responses to the trivalent inactivated seasonal influenza vaccine (TIV) [85], while women produce higher levels of antibodies than men in immunological responses to infections and vaccinations [86].

### 3.2. T Lymphocytes

Mature T cells do not express ARs on their surface, which makes them not sensitive to exogenous androgen modulation effects [30]. In contrast, both classical ARs, are expressed on the surface of CD4^+^CD8^+^ T cellular subsets [76]. In addition, double-negative (DN) and double-positive (DP) T cells also express classical ARs [38], as do thymic epithelial cells [87] and BM stromal cells [16]. This evidence highlights a theoretical androgen-modulation effect on the development and maturation of T cells. ER α in T cells suppresses the responses of follicular helper T cells and prevents autoimmunity. [88]. Estrogen modulates all subsets of T cells that include CD4^+^ (Th1, Th2, Th17, and Tregs) and CD8^+^ cells [75]. The expression of the PRα was upregulated in the luteal phase of the menstrual cycle in CD8^+^ but not in CD4^+^ [89].

Androgens influence T cell subpopulations in both women and men. Healthy men undergoing gonadal castration (via GnRH agonists administration) show a reduction in CD4^+^CD25^+^ T cell subpopulations, in comparison to untreated controls [90] (Table 1). Postmenopausal women with adrenal androgen deficiency and subjected to DHEA administration show a decrease in CD4^+^ and an increase in CD8^+^ T cells [38] (Table 1). Th1-type immune T-cell responses (pro-inflammatory) are more abundant in women, with respect to Th2-type responses (anti-inflammatory), which are expressed more in men [91]. Clinical evidence shows how females are afflicted with autoimmune T cell-mediated diseases (e.g., multiple sclerosis, rheumatoid arthritis, systemic lupus erythematosus) more frequently than men [3], pointing to a theoretical androgen influence as a protective factor against autoimmune diseases. The influence of androgens on autoimmune regulatory gene transcription (AIRE), which provides strong protection against autoimmunity, is stronger in men than in women [92]. In addition, T regulatory cells (T-regs), a T cell subpopulation tasked with immune system modulation in keeping anti-antigen tolerance and thus preventing autoimmune diseases [93], are also markedly higher in men [39] (Table 1). Women affected by adrenal insufficiency and who are subjected to in vivo androgen supplementation show an increase in T-regs number [40]. Upon further study, the FOXP3 transcription factor, a regulator of T-reg function and cell development, was examined and it was shown that Te treatment enhanced FOXP3 expression, thus exerting an androgen-mediated influence on T-regs [41].

## 4. Conclusions

The role of androgens is complex due to an intricate crosstalk with multiple organ systems, cellular processes, and metabolism into various bioactive compounds. The data discussed in this review support gender differences in the immune system that make men more prone to risk of microbial infections and less proficient in virus clearing, although more protected against autoimmune diseases, with respect to women. The level of male physiological androgens constitutes one of the factors contributing to establishing these sex differences. In fact, it was demonstrated that androgen hormones have been shown to affect more AR-positive immune cells. However, androgens also influence AR-negative cells, suggesting the existence of secondary mechanisms. The differential effects of testosterone on the circulation of neutrophils and monocytes and not on other cell types have been highlighted.

Since neutrophils and monocytes all derive from a common myeloid ancestor within the bone marrow, the selective upregulation of these cell types suggests that testosterone probably promotes the differentiation of a multipotential hematopoietic progenitor in the myeloid lineage.

Although the detected androgenic effects vary according to the different cells and different hormone levels, a relevant anti-inflammatory effect on immune cells was demonstrated. However, this androgenic influence is only one of the many factors that contribute to the immune response bias between men and women. The influence of immune-modulation E2 hormones should be considered. More attention should be given to genetic expression, a factor that must be analyzed due to its importance in the sex bias determination of immune cells. However, it should be noted that gender differences in immune cell function represent a relatively novel field, with many open questions that necessitate attention and further research. 

Overall, these findings highlight the great complexity of the topic, and that there are secondary mechanisms that need to be analyzed and deepened to dissect such biological networks. The current androgen replacement therapy protocols and their therapeutical implications also require further investigation to examine the immunological response.

## Data Availability

Not applicable.

## Abbreviations

Δ^4^: Δ^4^-androstenedione; ADT: Androgen deprivation therapy; AR: Androgen receptor; BM: Bone marrow; Cyt c: Cytochrome c; DCs: Dendritic cells; DHEA-S: Dehydroepiandrosterone sulfate; DHT: Dihydrotestosterone; dNKs: Decidual NKs; E_2_: Estradiol; GH: Growth hormone; hCG: Human chorionic gonadotropin; IFN-α: Interferon alpha; IFN-γ: Interferon gamma; IL-1β: Interleukin 1 beta; IL-6: Interleukin 6; IL-10: Interleukin 10; IL-12: Interleukin 12; LGL: Large granular lymphocytes cells; LH: Luteinizing hormone; LH/CGR: LH/CG receptor; MCs: Mast cells; mDCs: Myeloid-derived DCs; NET: Neutrophil extracellular traps; NKs: Natural killer cells; P_4_: Progesterone; PCOS: Polycystic ovary syndrome; pDCs: Peripheral dendritic cells; ROIs: Reactive oxygen intermediates; SHBG: Sex hormone-binding globulin protein; Te: Testosterone; T2DM: Type II diabetes mellitus; TNF-α: Tumor Necrosis Factor alpha.

## References

[1] Ortona E., Pierdominici M., Rider V. (2019). Editorial: Sex Hormones and Gender Differences in Immune Responses. Front. Immunol..

[2] Shepherd R., Cheung A.S., Pang K., Saffery R., Novakovic B. (2020). Sexual Dimorphism in Innate Immunity: The Role of Sex Hormones and Epigenetics. Front. Immunol..

[3] Henze L., Schwinge D., Schramm C. (2020). The Effects of Androgens on T Cells: Clues to Female Predominance in Autoimmune Liver Diseases?. Front. Immunol..

[4] Taneja V. (2018). Sex Hormones Determine Immune Response. Front. Immunol..

[5] Moulton V.R. (2018). Sex Hormones in Acquired Immunity and Autoimmune Disease. Front. Immunol..

[6] Rosato E., Sciarra F., Anastasiadou E., Lenzi A., Venneri M.A. (2022). Revisiting the physiological role of androgens in women. Expert Rev. Endocrinol. Metab..

[7] Sciarra F., Franceschini E., Campolo F., Gianfrilli D., Pallotti F., Paoli D., Isidori A.M., Venneri M.A. (2020). Disruption of Circadian Rhythms: A Crucial Factor in the Etiology of Infertility. Int. J. Mol. Sci..

[8] Baldassarri M., Picchiotti N., Fava F., Fallerini C., Benetti E., Daga S., Valentino F., Doddato G., Furini S., Giliberti A. (2021). Shorter androgen receptor polyQ alleles protect against life-threatening COVID-19 disease in European males. EBioMedicine.

[9] Rastrelli G., Di Stasi V., Inglese F., Beccaria M., Garuti M., Di Costanzo D., Spreafico F., Greco G.F., Cervi G., Pecoriello A. (2021). Low testosterone levels predict clinical adverse outcomes in SARS-CoV-2 pneumonia patients. Andrology.

[10] Mendes L.O., Castilho A.C.S., Pinho C.F., Gonçalvez B.F., Razza E.M., Chuffa L.G.A., Anselmo-Franci J.A., Scarano W.R., Martinez F.E. (2018). Modulation of inflammatory and hormonal parameters in response to testosterone therapy: Effects on the ventral prostate of adult rats. Cell Biol. Int..

[11] Lucas-Herald A.K., Touyz R.M. (2022). Androgens and Androgen Receptors as Determinants of Vascular Sex Differences Across the Lifespan. Can. J. Cardiol..

[12] Michmerhuizen A.R., Spratt D.E., Pierce L.J., Speers C.W. (2020). ARe we there yet? Understanding androgen receptor signaling in breast cancer. NPJ Breast Cancer.

[13] Bouman A., Heineman M.J., Faas M.M. (2005). Sex hormones and the immune response in humans. Hum. Reprod. Update.

[14] Cooke P.S., Walker W.H. (2022). Nonclassical androgen and estrogen signaling is essential for normal spermatogenesis. Semin Cell Dev. Biol..

[15] Becerra-Diaz M., Song M., Heller N. (2020). Androgen and Androgen Receptors as Regulators of Monocyte and Macrophage Biology in the Healthy and Diseased Lung. Front. Immunol..

[16] Mantalaris A., Panoskaltsis N., Sakai Y., Bourne P., Chang C., Messing E.M., Wu J.H. (2001). Localization of androgen receptor expression in human bone marrow. J. Pathol..

[17] Chakraborty B., Byemerwa J., Krebs T., Lim F., Chang C.Y., McDonnell D.P. (2023). Estrogen Receptor Signaling in the Immune System. Endocr. Rev..

[18] Tang Z.R., Zhang R., Lian Z.X., Deng S.L., Yu K. (2019). Estrogen-Receptor Expression and Function in Female Reproductive Disease. Cells.

[19] Solano M.E., Arck P.C. (2019). Steroids, Pregnancy and Fetal Development. Front. Immunol..

[20] Luetjens C.M., Didolkar A., Kliesch S., Paulus W., Jeibmann A., Böcker W., Nieschlag E., Simoni M. (2006). Tissue expression of the nuclear progesterone receptor in male non-human primates and men. J. Endocrinol..

[21] Jaillon S., Berthenet K., Garlanda C. (2019). Sexual Dimorphism in Innate Immunity. Clin. Rev. Allergy Immunol..

[22] Azeez J.M., Susmi T.R., Remadevi V., Ravindran V., Sasikumar Sujatha A., Ayswarya R.N.S., Sreeja S. (2021). New insights into the functions of progesterone receptor (PR) isoforms and progesterone signaling. Am. J. Cancer Res..

[23] Molloy E.J., O’Neill A.J., Grantham J.J., Sheridan-Pereira M., Fitzpatrick J.M., Webb D.W., Watson R.W. (2003). Sex-specific alterations in neutrophil apoptosis: The role of estradiol and progesterone. Blood.

[24] Blazkova J., Gupta S., Liu Y., Gaudilliere B., Ganio E.A., Bolen C.R., Saar-Dover R., Fragiadakis G.K., Angst M.S., Hasni S. (2017). Multicenter Systems Analysis of Human Blood Reveals Immature Neutrophils in Males and During Pregnancy. J. Immunol..

[25] El Kebir D., Filep J.G. (2013). Modulation of Neutrophil Apoptosis and the Resolution of Inflammation through β2 Integrins. Front. Immunol..

[26] Lentz L.S., Stutz A.J., Meyer N., Schubert K., Karkossa I., von Bergen M., Zenclussen A.C., Schumacher A. (2022). Human chorionic gonadotropin promotes murine Treg cells and restricts pregnancy-harmful proinflammatory Th17 responses. Front. Immunol..

[27] Schumacher A., Zenclussen A.C. (2019). Human Chorionic Gonadotropin-Mediated Immune Responses That Facilitate Embryo Implantation and Placentation. Front. Immunol..

[28] Markman J.L., Porritt R.A., Wakita D., Lane M.E., Martinon D., Noval Rivas M., Luu M., Posadas E.M., Crother T.R., Arditi M. (2020). Loss of testosterone impairs anti-tumor neutrophil function. Nat. Commun..

[29] Corrales J.J., Almeida M., Miralles J.M., Orfao A. (2009). Persistence of androgenic effects on the production of proinflammatory cytokines by circulating antigen-presenting cells after withdrawal of testosterone treatment in aging type 2 diabetic men with partial androgen deficiency. Fertil. Steril..

[30] Gagliano-Jucá T., Pencina K.M., Guo W., Li Z., Huang G., Basaria S., Bhasin S. (2020). Differential effects of testosterone on circulating neutrophils, monocytes, and platelets in men: Findings from two trials. Andrology.

[31] Batty M.J., Chabrier G., Sheridan A., Gage M.C. (2021). Metabolic Hormones Modulate Macrophage Inflammatory Responses. Cancers.

[32] Becerra-Díaz M., Strickland A.B., Keselman A., Heller N.M. (2018). Androgen and Androgen Receptor as Enhancers of M2 Macrophage Polarization in Allergic Lung Inflammation. J. Immunol..

[33] Wang J.P., Zhang L., Madera R.F., Woda M., Libraty D.H. (2012). Plasmacytoid dendritic cell interferon-α production to R-848 stimulation is decreased in male infants. BMC Immunol..

[34] Corrales J.J., Almeida M., Cordero M., Martín-Martín L., Méndez C., Miralles J.M., Orfao A. (2012). Enhanced immunological response by dendritic cells in male hypogonadism. Eur. J. Clin. Investig..

[35] Lundell A.C., Nordström I., Andersson K., Strömbeck A., Ohlsson C., Tivesten Å., Rudin A. (2017). Dihydrotestosterone levels at birth associate positively with higher proportions of circulating immature/naïve CD5(+) B cells in boys. Sci. Rep..

[36] Lundell A.C., Hesselmar B., Nordström I., Adlerberth I., Wold A.E., Rudin A. (2015). Higher B-cell activating factor levels at birth are positively associated with maternal dairy farm exposure and negatively related to allergy development. J. Allergy Clin. Immunol..

[37] Liu C.Y., Chang T.C., Lin S.H., Tsao C.W. (2022). Is a Ketogenic Diet Superior to a High-Fat, High-Cholesterol Diet Regarding Testicular Function and Spermatogenesis?. Front. Nutr..

[38] Tang J., Chen L.R., Chen K.H. (2021). The Utilization of Dehydroepiandrosterone as a Sexual Hormone Precursor in Premenopausal and Postmenopausal Women: An Overview. Pharmaceuticals.

[39] Afshan G., Afzal N., Qureshi S. (2012). CD4+CD25(hi) regulatory T cells in healthy males and females mediate gender difference in the prevalence of autoimmune diseases. Clin. Lab..

[40] Rutkowsky J.M., Knotts T.A., Ono-Moore K.D., McCoin C.S., Huang S., Schneider D., Singh S., Adams S.H., Hwang D.H. (2014). Acylcarnitines activate proinflammatory signaling pathways. Am. J. Physiol. Endocrinol. Metab..

[41] Walecki M., Eisel F., Klug J., Baal N., Paradowska-Dogan A., Wahle E., Hackstein H., Meinhardt A., Fijak M. (2015). Androgen receptor modulates Foxp3 expression in CD4+CD25+Foxp3+ regulatory T-cells. Mol. Biol Cell.

[42] Tang Q., Cheng B., Dai R., Wang R. (2021). The Role of Androgen Receptor in Cross Talk Between Stromal Cells and Prostate Cancer Epithelial Cells. Front. Cell Dev. Biol..

[43] Morris M.J., Mota J.M., Lacuna K., Hilden P., Gleave M., Carducci M.A., Saad F., Cohn E.D., Filipenko J., Heller G. (2021). Phase 3 Randomized Controlled Trial of Androgen Deprivation Therapy with or Without Docetaxel in High-risk Biochemically Recurrent Prostate Cancer After Surgery (TAX3503). Eur. Urol. Oncol..

[44] Marin D.P., Bolin A.P., dos Santos Rde C., Curi R., Otton R. (2010). Testosterone suppresses oxidative stress in human neutrophils. Cell Biochem. Funct..

[45] Traish A., Bolanos J., Nair S., Saad F., Morgentaler A. (2018). Do Androgens Modulate the Pathophysiological Pathways of Inflammation? Appraising the Contemporary Evidence. J. Clin. Med..

[46] Szczepanek-Parulska E., Adamska M., Korda O., Kosicka W., Skowrońska D., Świejkowska A., Tuzimek D., Dadej D., Krygier A., Ruchała M. (2021). Changes in complete blood count parameters influenced by endocrine disorders. Endokrynol. Pol..

[47] Ibáñez L., Valls C., de Zegher F. (2006). Discontinuous low-dose flutamide-metformin plus an oral or a transdermal contraceptive in patients with hyperinsulinaemic hyperandrogenism: Normalizing effects on CRP, TNF-alpha and the neutrophil/lymphocyte ratio. Hum. Reprod..

[48] Dama A., Baggio C., Boscaro C., Albiero M., Cignarella A. (2021). Estrogen Receptor Functions and Pathways at the Vascular Immune Interface. Int. J. Mol. Sci..

[49] McGlade E.A., Miyamoto A., Winuthayanon W. (2022). Progesterone and Inflammatory Response in the Oviduct during Physiological and Pathological Conditions. Cells.

[50] Consiglio C.R., Gollnick S.O. (2020). Androgen Receptor Signaling Positively Regulates Monocytic Development. Front. Immunol..

[51] Posma E., Moes H., Heineman M.J., Faas M.M. (2004). The effect of testosterone on cytokine production in the specific and non-specific immune response. Am. J. Reprod. Immunol..

[52] Bhasin S., Apovian C.M., Travison T.G., Pencina K., Moore L.L., Huang G., Campbell W.W., Li Z., Howland A.S., Chen R. (2018). Effect of Protein Intake on Lean Body Mass in Functionally Limited Older Men: A Randomized Clinical Trial. JAMA Intern. Med..

[53] Bhasin S., Travison T.G., Storer T.W., Lakshman K., Kaushik M., Mazer N.A., Ngyuen A.H., Davda M.N., Jara H., Aakil A. (2012). Effect of testosterone supplementation with and without a dual 5alpha-reductase inhibitor on fat-free mass in men with suppressed testosterone production: A randomized controlled trial. JAMA.

[54] O’Connor M.F., Motivala S.J., Valladares E.M., Olmstead R., Irwin M.R. (2007). Sex differences in monocyte expression of IL-6: Role of autonomic mechanisms. Am. J. Physiol. Regul. Integr. Comp. Physiol..

[55] Cioni B., Zaalberg A., van Beijnum J.R., Melis M.H.M., van Burgsteden J., Muraro M.J., Hooijberg E., Peters D., Hofland I., Lubeck Y. (2020). Androgen receptor signalling in macrophages promotes TREM-1-mediated prostate cancer cell line migration and invasion. Nat. Commun..

[56] Dressing G.E., Goldberg J.E., Charles N.J., Schwertfeger K.L., Lange C.A. (2011). Membrane progesterone receptor expression in mammalian tissues: A review of regulation and physiological implications. Steroids.

[57] Lee G.T., Kim J.H., Kwon S.J., Stein M.N., Hong J.H., Nagaya N., Billakanti S., Kim M.M., Kim W.J., Kim I.Y. (2019). Dihydrotestosterone Increases Cytotoxic Activity of Macrophages on Prostate Cancer Cells via TRAIL. Endocrinology.

[58] Godoy-Pacheco A., García-Chagollán M., Ramírez-De-Arellano A., Hernández-Silva C.D., Villegas-Pineda J.C., Ramírez-López I.G., Zepeda-Nuño J.S., Aguilar-Lemarroy A., Pereira-Suárez A.L. (2022). Differential modulation of natural killer cell cytotoxicity by 17β-estradiol and prolactin through the NKG2D/NKG2DL axis in cervical cancer cells. Oncol. Lett..

[59] Rabijewski M., Papierska L., Binkowska M., Maksym R., Jankowska K., Skrzypulec-Plinta W., Zgliczynski W. (2020). Supplementation of dehydroepiandrosterone (DHEA) in pre- and postmenopausal women—Position statement of expert panel of Polish Menopause and Andropause Society. Ginekol. Pol..

[60] Mahajan D., Sharma N.R., Kancharla S., Kolli P., Tripathy A., Sharma A.K., Singh S., Kumar S., Mohanty A.K., Jena M.K. (2022). Role of Natural Killer Cells during Pregnancy and Related Complications. Biomolecules.

[61] Wallace A.E., Fraser R., Cartwright J.E. (2012). Extravillous trophoblast and decidual natural killer cells: A remodelling partnership. Hum. Reprod. Update.

[62] Fraser R., Zenclussen A.C. (2022). Killer Timing: The Temporal Uterine Natural Killer Cell Differentiation Pathway and Implications for Female Reproductive Health. Front. Endocrinol..

[63] Raghupathy R., Szekeres-Bartho J. (2022). Progesterone: A Unique Hormone with Immunomodulatory Roles in Pregnancy. Int. J. Mol. Sci..

[64] Vom Steeg L.G., Dhakal S., Woldetsadik Y.A., Park H.S., Mulka K.R., Reilly E.C., Topham D.J., Klein S.L. (2020). Androgen receptor signaling in the lungs mitigates inflammation and improves the outcome of influenza in mice. PLoS Pathog..

[65] Watanabe Y., Tajiki-Nishino R., Tajima H., Fukuyama T. (2019). Role of estrogen receptors α and β in the development of allergic airway inflammation in mice: A possible involvement of interleukin 33 and eosinophils. Toxicology.

[66] Artham S., Chang C.Y., McDonnell D.P. (2023). Eosinophilia in cancer and its regulation by sex hormones. Trends Endocrinol. Metab..

[67] Similowski T., Orcel B., Derenne J.P. (1997). CD8+ lymphocytic pneumonitis in a patient receiving cyproterone acetate. South. Med. J..

[68] Xiong Y.L., Liang X.Y., Yang X., Li Y., Wei L.N. (2011). Low-grade chronic inflammation in the peripheral blood and ovaries of women with polycystic ovarian syndrome. Eur. J. Obstet. Gynecol. Reprod. Biol..

[69] Zaitsu M., Narita S., Lambert K.C., Grady J.J., Estes D.M., Curran E.M., Brooks E.G., Watson C.S., Goldblum R.M., Midoro-Horiuti T. (2007). Estradiol activates mast cells via a non-genomic estrogen receptor-alpha and calcium influx. Mol. Immunol..

[70] Chen W., Beck I., Schober W., Brockow K., Effner R., Buters J.T., Behrendt H., Ring J. (2010). Human mast cells express androgen receptors but treatment with testosterone exerts no influence on IgE-independent mast cell degranulation elicited by neuromuscular blocking agents. Exp. Dermatol..

[71] Muñoz-Cruz S., Mendoza-Rodríguez Y., Nava-Castro K.E., Yepez-Mulia L., Morales-Montor J. (2015). Gender-related effects of sex steroids on histamine release and FcεRI expression in rat peritoneal mast cells. J. Immunol. Res..

[72] Mackey E., Thelen K.M., Bali V., Fardisi M., Trowbridge M., Jordan C.L., Moeser A.J. (2020). Perinatal androgens organize sex differences in mast cells and attenuate anaphylaxis severity into adulthood. Proc. Natl. Acad. Sci. USA.

[73] Salvati L., Vitiello G., Parronchi P. (2019). Gender differences in anaphylaxis. Curr. Opin. Allergy Clin. Immunol..

[74] Paharkova-Vatchkova V., Maldonado R., Kovats S. (2004). Estrogen preferentially promotes the differentiation of CD11c+ CD11b(intermediate) dendritic cells from bone marrow precursors. J. Immunol..

[75] Khan D., Ansar Ahmed S. (2015). The Immune System Is a Natural Target for Estrogen Action: Opposing Effects of Estrogen in Two Prototypical Autoimmune Diseases. Front. Immunol..

[76] Ben-Batalla I., Vargas-Delgado M.E., von Amsberg G., Janning M., Loges S. (2020). Influence of Androgens on Immunity to Self and Foreign: Effects on Immunity and Cancer. Front. Immunol..

[77] Corrales J.J., Almeida M., Martín-Martín L., Miralles J.M., Orfao A. (2014). Testosterone replacement therapy in hypogonadal men is associated with increased expression of LAMP-2 (CD107b) by circulating monocytes and dendritic cells. Clin. Endocrinol..

[78] Berghöfer B., Frommer T., Haley G., Fink L., Bein G., Hackstein H. (2006). TLR7 ligands induce higher IFN-alpha production in females. J. Immunol..

[79] Psarras A., Alase A., Antanaviciute A., Carr I.M., Md Yusof M.Y., Wittmann M., Emery P., Tsokos G.C., Vital E.M. (2020). Functionally impaired plasmacytoid dendritic cells and non-haematopoietic sources of type I interferon characterize human autoimmunity. Nat. Commun..

[80] Guéry J.C. (2021). Sex Differences in Primary HIV Infection: Revisiting the Role of TLR7-Driven Type 1 IFN Production by Plasmacytoid Dendritic Cells in Women. Front. Immunol..

[81] Fink A.L., Klein S.L. (2018). The evolution of greater humoral immunity in females than males: Implications for vaccine efficacy. Curr. Opin. Physiol..

[82] Martini E., Giugliano S., Rescigno M., Kallikourdis M. (2020). Regulatory T Cells Beyond Autoimmunity: From Pregnancy to Cancer and Cardiovascular Disease. Front. Immunol..

[83] Liu N., Yan W., Su R., Zhang L., Wang X., Li Z., Qin D., Peng J. (2022). Research progress on rheumatoid arthritis-associated depression. Front. Behav. Neurosci..

[84] Saudi A., Banday V., Zirakzadeh A.A., Selinger M., Forsberg J., Holmbom M., Henriksson J., Waldén M., Alamdari F., Aljabery F. (2023). Immune-Activated B Cells Are Dominant in Prostate Cancer. Cancers.

[85] Furman D., Hejblum B.P., Simon N., Jojic V., Dekker C.L., Thiébaut R., Tibshirani R.J., Davis M.M. (2014). Systems analysis of sex differences reveals an immunosuppressive role for testosterone in the response to influenza vaccination. Proc. Natl. Acad. Sci. USA.

[86] Wen S., Wu Z., Zhong S., Li M., Shu Y. (2021). Factors influencing the immunogenicity of influenza vaccines. Hum. Vaccin Immunother..

[87] Lai K.P., Lai J.J., Chang P., Altuwaijri S., Hsu J.W., Chuang K.H., Shyr C.R., Yeh S., Chang C. (2013). Targeting thymic epithelia AR enhances T-cell reconstitution and bone marrow transplant grafting efficacy. Mol. Endocrinol..

[88] Kim D.H., Park H.J., Park H.S., Lee J.U., Ko C., Gye M.C., Choi J.M. (2019). Estrogen receptor α in T cells suppresses follicular helper T cell responses and prevents autoimmunity. Exp. Mol. Med..

[89] Dosiou C., Hamilton A.E., Pang Y., Overgaard M.T., Tulac S., Dong J., Thomas P., Giudice L.C. (2008). Expression of membrane progesterone receptors on human T lymphocytes and Jurkat cells and activation of G-proteins by progesterone. J. Endocrinol..

[90] Page S.T., Plymate S.R., Bremner W.J., Matsumoto A.M., Hess D.L., Lin D.W., Amory J.K., Nelson P.S., Wu J.D. (2006). Effect of medical castration on CD4+ CD25+ T cells, CD8+ T cell IFN-gamma expression, and NK cells: A physiological role for testosterone and/or its metabolites. Am. J. Physiol. Endocrinol. Metab..

[91] Massa M.G., David C., Jörg S., Berg J., Gisevius B., Hirschberg S., Linker R.A., Gold R., Haghikia A. (2017). Testosterone Differentially Affects T Cells and Neurons in Murine and Human Models of Neuroinflammation and Neurodegeneration. Am. J. Pathol..

[92] Berrih-Aknin S., Panse R.L., Dragin N. (2018). AIRE: A missing link to explain female susceptibility to autoimmune diseases. Ann. N. Y. Acad. Sci..

[93] Goswami T.K., Singh M., Dhawan M., Mitra S., Emran T.B., Rabaan A.A., Mutair A.A., Alawi Z.A., Alhumaid S., Dhama K. (2022). Regulatory T cells (Tregs) and their therapeutic potential against autoimmune disorders—Advances and challenges. Hum. Vaccin Immunother..

